# Editorialets

**Published:** 1910-02

**Authors:** 


### Editorialets.

---

THE EDITOR of one of the Tentacles, in a recent number, said "if he hadn't went." Professor Keene will take notice.

---

THE *Texas State Journal of Medicine,* heretofore printed by the Von Boeckmann-Jones Printing Company at Austin, will hereafter be printed at Fort Worth.

---

DEAR DR. DANIEL: I hope you will not let up on your preachment of ethical truths in general. I always value anything you say in that way, and I am sure many others do.—*Geo. Dock.*

---

MISE EN SCENE: Some young mice were nibbling at the "scenery" in the deserted theater. Mother mouse passed by when the baby mouse remarked, "Hear me gnaw, ma." [Now, see some smarty say "Daniel spells 'mice' with an 's.' "]

---

ASTRONOMICAL.—The girl Saturn a rose-colored couch. The pale medical student paused and looked in. "Come in and spend an hour with Venus," she said. "No, thanks. I spent an hour with Venus last year already, and then I spent six months with Mercury, at the hospital. Ever since then I have been laid up at Ma's. It is a Sirius matter, don't Juno." And he hove a sigh and moseyed on.

---

THE case of J. M. Newman, appealed from Kendall county, was affirmed yesterday by the Court of Criminal Appeals. In this case the appellant was convicted and sentenced to one hour in jail and to pay a fine of $50 for practicing medicine without a license. Newman is a masseur.

---

THE FOLLOWING PRAYER was a great favorite with the late great Governor, John Albert Johnson, of Minnesota. It is worth preserving:

"Spare me from the bitterness and the sharp passions of unguarded moments. May I not forget that poverty and riches are of the spirit. * * * And, although age and infirmity overtake me, and I come not within sight of the castle of my dreams, teach me to be thankful for life, and for time's olden memories that are good and sweet, and may the evening's twilight find me gentle still."

---

ALAS, for the decay of English grammar. Philmil-Jones-the-Joke, Captain of The Hosts on the Golden Horn, Keeper of the Record and Seals, and chief scribe of the California Tentacle of The Octopus, says, editorially: "The people should be told the real facts." Good idea. They might get hold of some "facts" that are not "real." Professor Keene should take notice and write to The Octopus about it. The literary critic of a New York publication,—one who passes on papers,—writes, editorially: "We recommend those whom [sic] we think can serve them." The writers who say "between you and I," and "those kind," and "the most perfect," and "quite a few" are as thick as blackberries in a briar patch. What's the use?

